# Supplementary material for: Tissue-Specific Transcriptome and Metabolome Analysis Reveals the Response Mechanism of Brassica napus to Waterlogging Stress
Source: Int J Mol Sci. 2023 Mar 23;24(7):6015. doi: 10.3390/ijms24076015 (PMC10094381; doi:10.3390/ijms24076015)
Supplement: Supplementary file 1 [file ijms-24-06015-s001.zip › Supplementary figures.pdf]

Supporting Information

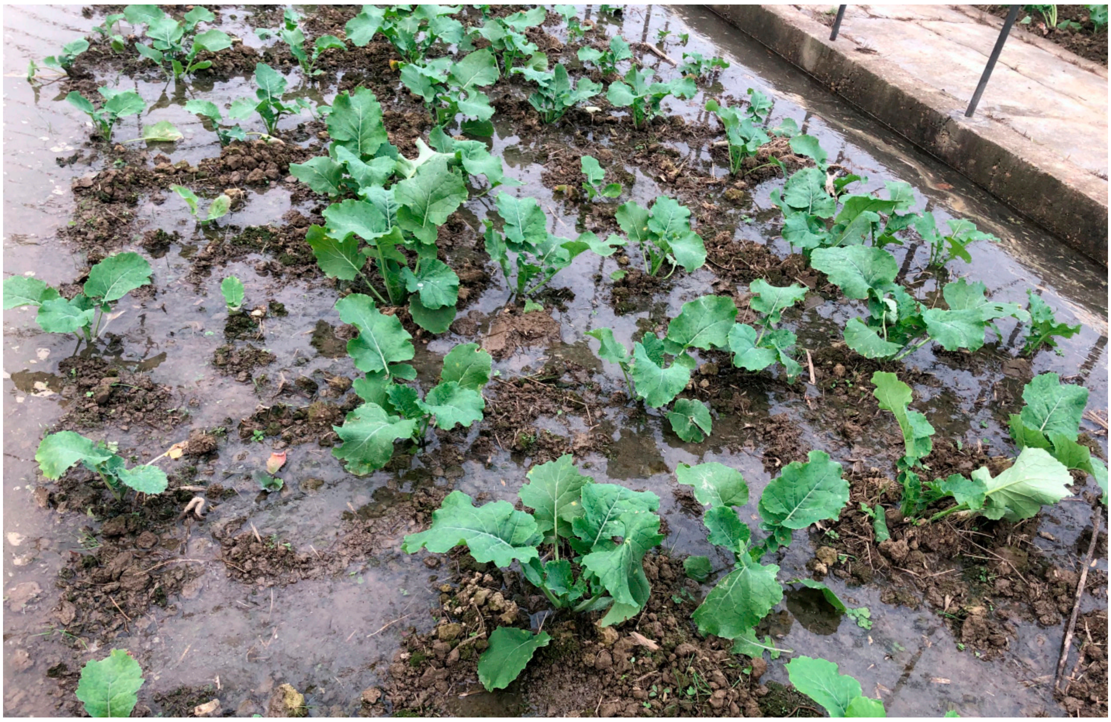

Figure S1. Field waterlogging stress. The field water content reached 90%.

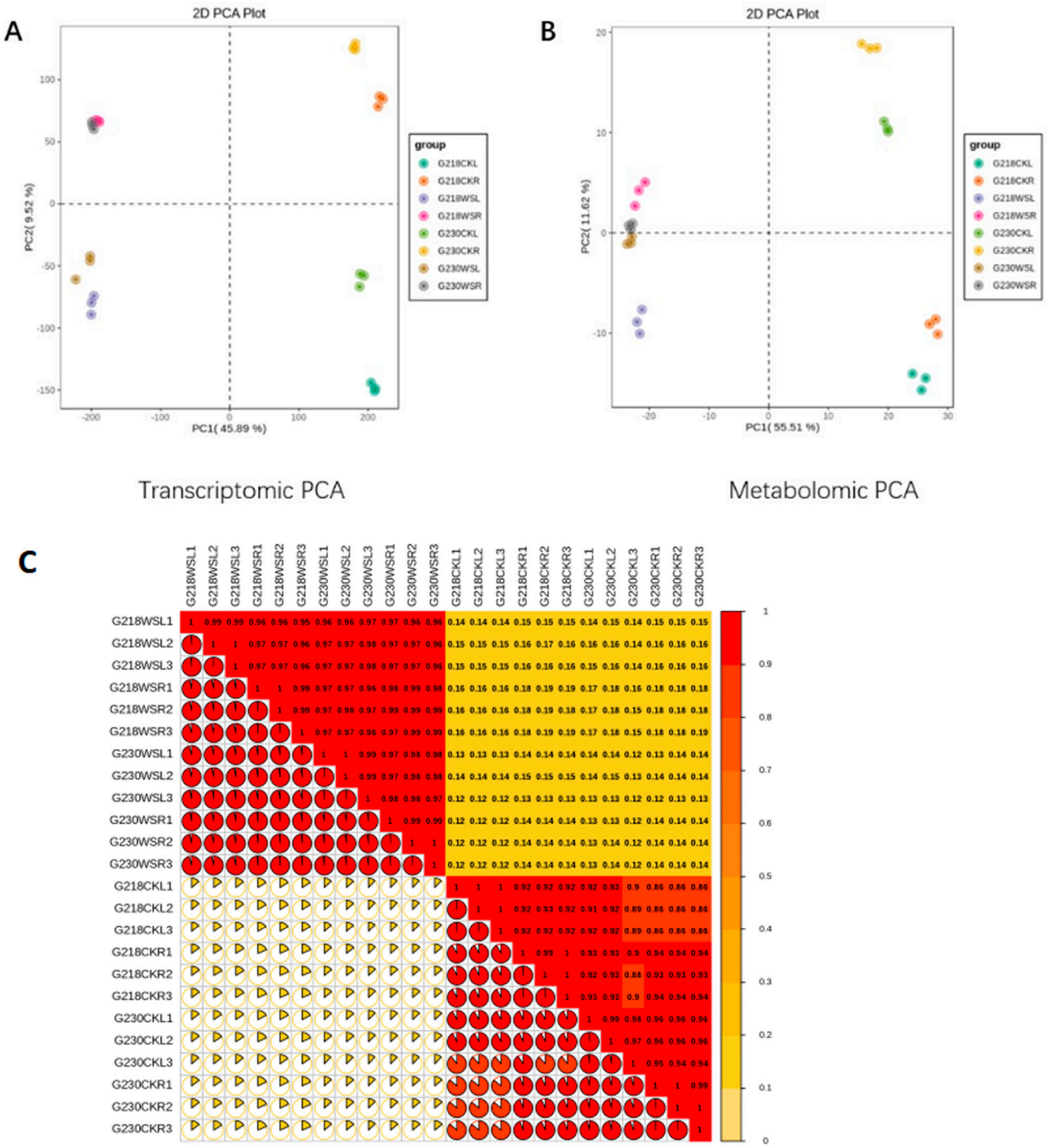

Figure S2. Sample PCA analysis and correlation analysis. (A) Transcriptomic PCA analysis. (B) Metabolomics PCA Analysis. (C) Transcriptomics sample correlation analysis heatmap.

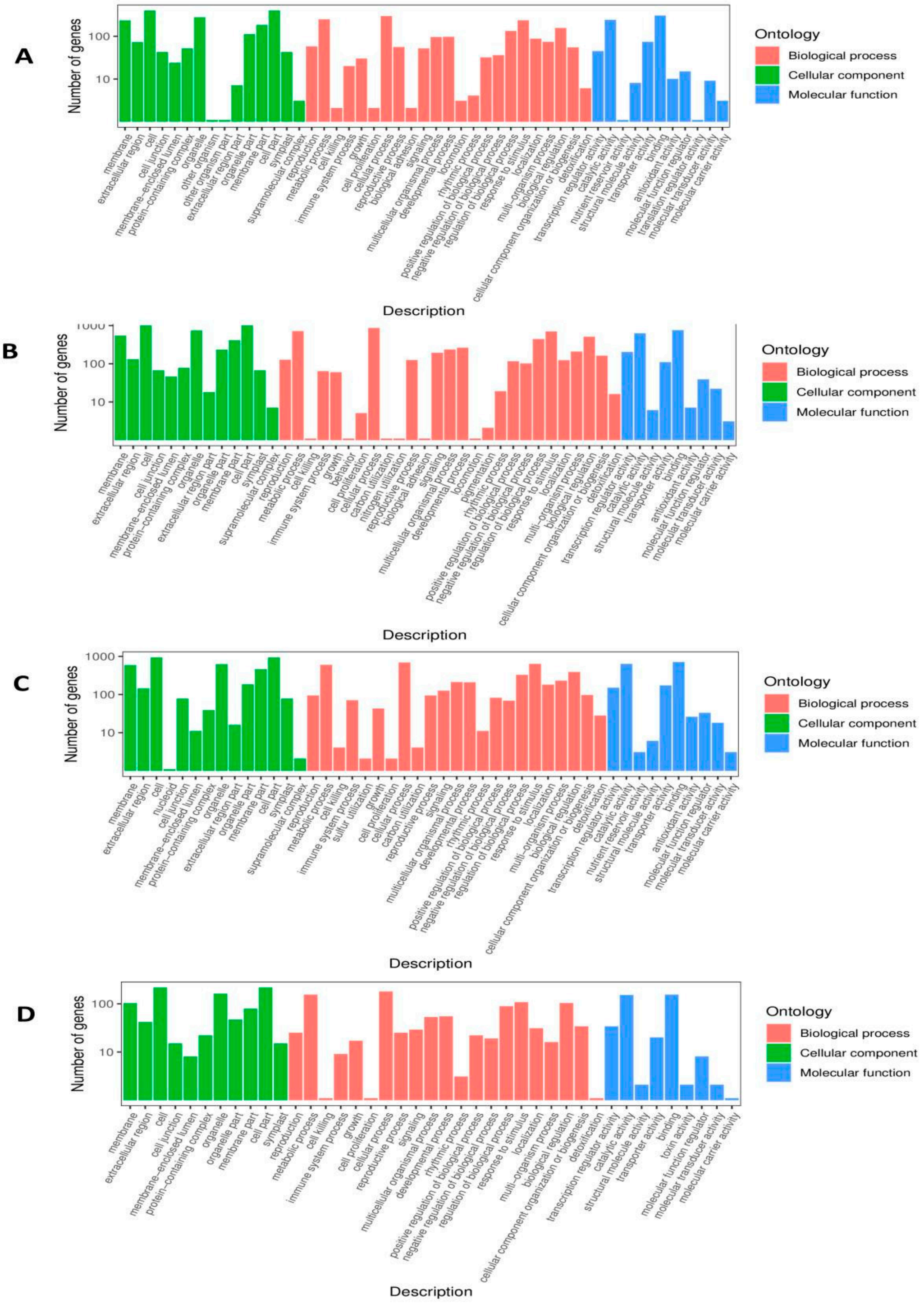

Figure S3. GO enrichment analysis of DEGs in different comparative combinations under waterlogging stress. (A) Top 50 GO Terms with the most enriched DEGs in the G218CKL_vs_G230CKL comparison. (B) Top 50 GO Terms with the most enriched DEGs in the G218WSL_vs_G230WSL comparison. (C) Top 50 GO Terms with the most enriched DEGs in the G218CKR_vs_G230CKR comparison. (D) Top 50 GO Terms with the most enriched DEGs in the G218WSR_vs_G230WSR comparison.

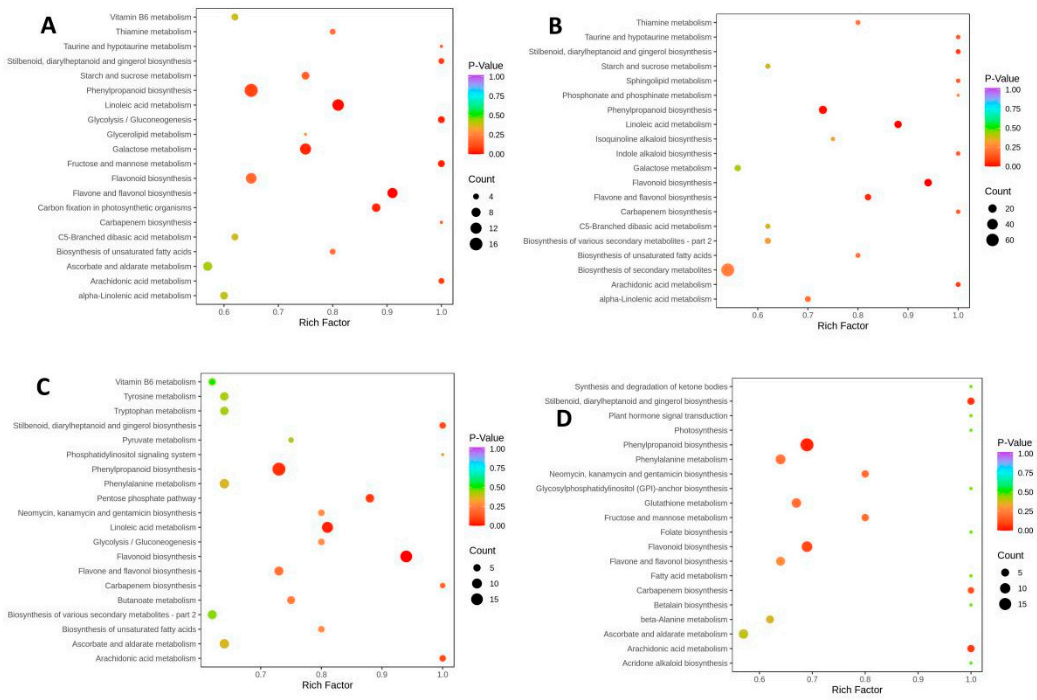

Figure S4. KEGG enrichment analysis of DEMs of different comparative combinations under waterlogging stress. (A) Significantly enriched KEGG pathways in the G218CKL vs. G218WSL comparison. (B) Significantly enriched KEGG pathways in the G218CKR vs. G218WSR comparison. (C) Significantly enrichted KEGG pathways in the G230CKL vs. G230WSL comparison. (D) Significantly enriched KEGG pathways in the G230CKR vs. G230WSR comparison.
