# Supplementary material for: Tissue-Specific Transcriptome and Metabolome Analysis Reveals the Response Mechanism of Brassica napus to Waterlogging Stress
Source: Int J Mol Sci. 2023 Mar 23;24(7):6015. doi: 10.3390/ijms24076015 (PMC10094381; doi:10.3390/ijms24076015)
Supplement: Supplementary file 1 [file ijms-24-06015-s001.zip › Table S1.pdf]

Table S1 Primers used for RT-qPCR

Gene name	Forward Primer (5′-3′)	Reverse Primer (5′-3′)	Product Size(bp)	TM(℃)
CHI (BnaA01G0107500ZS)	CCCGACCAGAGAGTTGAG	TGATGTAATGCGGCGTAA	154	53
DFR (BnaC09G0215200ZS)	AACGAAGTGATAAAACCA	TCCAGCAGACGAAGTAAA	102	49
PDX2 (BnaA02T0098300ZS)	TATCTCAGTGCCTCTTCT	CTTCCTCTTGGATTTGTA	176	49
CER26L(BnaC02G0495800ZS)	TACCCTCGTAGTCCATTC	TGATCTCAGCCACTCATC	148	52
Actin2.1	GGTTGGGATGGACCAGAAGG	TCAGGAGCAATACGGAGC	180	55
