# Supplementary material for: Tissue-Specific Transcriptome and Metabolome Analysis Reveals the Response Mechanism of Brassica napus to Waterlogging Stress
Source: Int J Mol Sci. 2023 Mar 23;24(7):6015. doi: 10.3390/ijms24076015 (PMC10094381; doi:10.3390/ijms24076015)
Supplement: Supplementary file 1 [file ijms-24-06015-s001.zip › Table S4.pdf]

Table S4 Dead seedling rate of 32 varieties or inbred lines of Brassica napus seedlings under waterlogging stress for 6 days and reoxygenation for 7 days

Code	Material	Death seedling	Average death rate(%)
1	G013	16	26.6
2	G036	23	38.3
3	G039	21	35
4	G051	18	30
5	G114	20	33.3
5	G179	3	5
6	G211	1	1.7
7	G218	60	100
8	G230	0	0
9	G233	58	96.7
10	G235	28	46.7
11	G264	17	28.3
12	G268	59	98.3
13	G344	20	33.3
14	G345	19	31.7
15	G346	16	26.7
16	G347	18	30
17	G02	48	80
18	Xiangyou15	8	13.3
19	WB06	17	28.3
20	708	21	35
21	710	16	26.7
22	G201	18	30
23	G209	36	60
24	G241	43	71.7
25	G250	9	15
26	G251	15	25
27	ZS11	11	18.3
28	Zheyou036	17	28.3
29	M004	22	36.7
30	98C40	16	26.7
31	104	13	21.7
32	1035	16	26.7
